# Prevalence and antifungal susceptibility profiles of *Candida* isolates among patients with candiduria: a multiplex PCR assay

**DOI:** 10.22034/cmm.2025.345248.1610

**Published:** 2025-08-03

**Authors:** Sima Darabian, Sepideh Pakravan, Manizhe Jozpanahi, Afsaneh Karami, Saeid Amanloo, Hamid Morovati

**Affiliations:** 1 Department of Medical Parasitology and Mycology, School of Medicine, Zanjan University of Medical Sciences, Zanjan, Iran; 2 Department of Infectious Diseases, Valiasr Hospital, Zanjan University of Medical Sciences, Zanjan, Iran; 3 Department of Parasitology and Mycology, Faculty of Medicine, Tabriz University of Medical Sciences, Tabriz, Iran

**Keywords:** Antifungal resistance, *Candida* species, Candiduria, Multiplex PCR, Susceptibility profiles

## Abstract

**Background and Purpose:**

Incidence of candiduria attributed to *Candida* species has been increasing, with a notable rise in cases involving antifungal-resistant non-*albicans Candida* (NAC) species. This investigation aimed to assess both the prevalence and antifungal susceptibility patterns of *Candida* isolates obtained from patients diagnosed with candiduria.

**Materials and Methods:**

In total, 100 urine specimens were collected from patients diagnosed with candiduria and subjected to analysis. Subsequent to the preliminary identification, a 21-plex polymerase chain reaction (PCR) assay was employed for species characterization. Antifungal susceptibility testing was conducted using the broth microdilution technique, which aimed to determine the minimum inhibitory concentrations (MICs) of fluconazole, amphotericin B, and caspofungin.

**Results:**

Among the 100 analyzed patients, *Candida albicans* was the predominant species, accounting for 70% of isolates, followed by *C. tropicalis* (11%), *C. glabrata* (9%), and *C. parapsilosis* (5%). Resistance to fluconazole was observed in 2.86% of *C. albicans* isolates, whereas 29.41% of the NAC species exhibited resistance to this antifungal agent.

**Conclusion:**

The fluconazole resistance rate was notably higher among NAC species, compared to that of *C. albicans*. To deepen current understanding, it is recommended that future molecular investigations employ advanced and diverse methodologies, along with larger and more representative patient cohorts.

## Introduction

Hospital-acquired infections (HAIs) represent a major contributor to patient mortality and are associated with a significant escalation in healthcare expenditures [ 1 ]. Infections caused by pathogenic fungi are among the main HAIs associated with high mortality and complications [ 2 ]. Recently, there has been a significant increase in patients hospitalized in intensive care units (ICUs), even in European and American countries [ 3 ]. Candidiasis is one of the most critical hospital-acquired fungal infections [ 4 ]. *Candida* yeast species are responsible for 10% of hospital-acquired bloodstream infections and rank as the fourth leading cause of these infections in the USA [ 5 ]. Moreover, *Candida* species cause more than 75% of nosocomial infections [ 5 ].

Candiduria is a urinary tract infection caused by *Candida* species and is among the most prevalent invasive fungal infections [ 6 ]. It can be classified as a HAI. However, it may result from cystitis, pyelonephritis, prostatitis, epididymal-orchitis, or disseminated candidiasis [ 6 ]. Diabetes, urinary catheters, use of broad-spectrum antibiotics, urinary obstruction, and ICU hospitalization are the main risk factors [ 6 ]. In most cases, the detection of *Candida* species in the urinary tract of asymptomatic individuals does not warrant clinical intervention, with the exception of specific high-risk groups, such as neutropenic patients, low birth weight neonates, and individuals undergoing urological procedures [ 6 , 7 ].

While *Candida albicans* remains the most frequently isolated species from urinary specimens, non-*albicans Candida* (NAC) species account for nearly half of urinary *Candida* isolates—a distribution that contrasts with the predominance of *C. albicans* observed in mucosal candidiasis. Other species include *C. glabrata* (25-30%), *C. tropicalis*, *C. krusei*, and *C. guilliermondii* (8-28%) [ 8 ].

One of the most challenging issues in the treatment of invasive fungal infections (IFIs) is accurate diagnosis [ 9 ]. Traditional methods, including clinical evaluation as well as culture, radiological, and histopathological evidence, have faced some obstacles, such as their high costs, time-consuming procedures, invasive interventions, and false positive and negative results [ 9 ]. These challenges caused scientists to pursue faster, more practical, and more reliable methods [ 9 ]. The polymerase chain reaction (PCR) method targets the biological characteristics of fungi and achieves faster results with higher specificity and sensitivity. In the plex PCR-21 method, a multiplex PCR system, specific gene regions for yeasts are targeted and replicated. The reaction is performed in three tubes using 21 primer pairs [ 10 - 13 ].

Determination of the *Candida* species causing the disease is of particular importance, since some species have inherent resistance while others can develop acquired resistance to the antifungal [ 14 , 15 ]. Furthermore, determination of the minimum inhibitory concentration (MIC) through the broth microdilution method and interpretation through the Clinical and Laboratory Standards Institute (CLSI) protocol is vital for the determination of clinically resistant isolates [ 16 ]. These factors are pivotal in guiding the selection of an appropriate therapeutic regimen for affected patients.

This study investigated the molecular epidemiology, through a 21-plex PCR system, and antifungal susceptibility profiles, through the broth microdilution method, of *Candida* species among patients with candiduria in Zanjan, northwestern Iran.

## Materials and Methods

### Ethics approval and consent to participate

This study was approved by the Ethics Committee of Zanjan University of Medical Science (Permission ID: IR.ZUMS.REC.1400.387). Informed consent was obtained from all participants, parents/legally authorized representatives of all minors (ages less than 16 years), and deceased participants. All methods were performed in accordance with the relevant guidelines and regulations (Declaration of Helsinki).

### Patients

This cross-sectional, single-center study was conducted on 120 inpatients at Valiasr Hospital in Zanjan, Iran, over a six-month period from January to July 2022. Twenty participants were missed, and 100 participants were included in further assays. Ethical approval for the present research was obtained from the Research Ethics Committee of Zanjan University of Medical Sciences, under the approval code IR.ZUMS.REC.1400.387. Written informed consent was acquired from patients. Relevant clinical and demographic information—including age, gender, surgical history, underlying comorbidities, reasons for hospitalization, duration of hospital stay, treatment modalities, and patient outcomes—was systematically collected and recorded when available. Inclusion of patients required specific clinical criteria indicative of candiduria, as defined in prior literature [ 17 ], and was guided by the clinical judgment of the attending physician. The distinction between *Candida* positive and negative was initially established by clinical signs and symptoms, including fever, as previously outlined [ 18 ]. Moreover, the yeast colony count test was performed through the colony-forming unit (CFU) technique to distinguish between colonization, normal yeast flora, and infection cases. The 10^3^ CFU/mL was considered a cutoff to distinguish between *Candida* colonization/normal flora and infection. The CFU counts below this threshold are considered colonization, while counts above it are more likely to indicate infection [ 19 ]. It should be mentioned that patients with unclear pre-hospitalization histories, chronic fungal infections, or unspecified antifungal treatment status were excluded from the study cohort.

### Samples and initial yeast isolation

Samples were collected from all hospital wards, including emergency, internal wards, urology, infectious diseases, ICU, surgery, cardiovascular, and neurology. In total, 100 urine samples were collected and immediately cultured on Sabouraud dextrose agar and Nutrient Agar (both from Merck, Germany). Cultures were then incubated at 35 °C for 24 h to facilitate fungal proliferation. Cultures with colonies equal to or more than 10^3^ were considered positive for *Candida* infection. Microscopic analysis was conducted using lactophenol cotton blue staining to detect fungal structures, including hyphae, pseudohyphae, and budding yeast cells, as well as to assess the presence of bacterial contamination. Afterward, for yeast purified colonies, yeast colonies were recultured on Sabouraud dextrose agar containing antibiotics (chloramphenicol and gentamicin) and incubated at 30 °C for 48 h (Supplementary Figure 1). In preparation for molecular assays and antifungal susceptibility tests (AFST), positive cells were moved to sterile standard saline tubes and kept at -20 °C.

### Molecular assays

### DNA extraction

Two mechanical (glass bead beating) [ 20 ] and chemical (phenol-chloroform) [ 21 ] methods were applied for DNA extraction. Accordingly, the following procedures were performed for DNA extraction: First, the glass beads were washed and dried three times. A loopful of yeast cells was resuspended in 200 µL of lysis buffer composed of 10 mM Tris (pH 8.0), 1 mM EDTA, 100 mM NaCl, 1% sodium dodecyl sulfate, and 2% Triton X-100. Subsequently, 300 µL of glass beads were added, and the cell mixture was further supplemented with 200 µL of a 1:1 phenol–chloroform solution. The cells were vortexed for 3 min, and 200 µL of TE, containing 10 mM Tris pH 8.0 and 1 mM EDTA, was added. The microtubes were centrifuged at 15,000 × g for 5 min, after which the aqueous phase was carefully transferred to a fresh microtube. Subsequently, 900 µL of ethanol was added to each sample to facilitate nucleic acid precipitation. The DNA was pelleted with centrifugation (5 min at 15,000 g). The resulting DNA pellet was washed once with 70% ethanol and subjected to centrifugation at 15,000 × g for 5 min. The purified DNA was then resuspended in 100 µL of TE buffer, and 1 µL of this solution was utilized for subsequent molecular analyses.

### Multiplex PCR assay

Initial molecular identification was carried out using a 21-plex PCR assay targeting the panfungal internal transcribed spacer (ITS) gene region. The procedure employed 21 specific primer pairs distributed across three separate reaction tubes, following previously established protocols [ 22 ]. First of all, the system was optimized by testing 21 standard isolates that were known previously (Supplementary Table 1). The first tube targeted *C. albicans*, *C. glabrata*, *C. parapsilosis*, *C. tropicalis*, *C. krusei*, *C. dubliniensis*, and *C. auris*. The second tube was targeted for *C. famata*, *C. rugosa*, *C. lusitaniae*, *C. guilliermondii*, *C. norvegensis*, *C. kefyr*, and *C. lipolytica*. The third tube was targeted for *Cryptococcus neoformans*, *Cryptococcus deneoformans*, *Cryptococcus gattii*, *Trichosporon asahii*, *Trichosporon lactis*, *Geotrichum candidum*, and *Rhodotorula mucilaginosa*.

**Figure 1:**
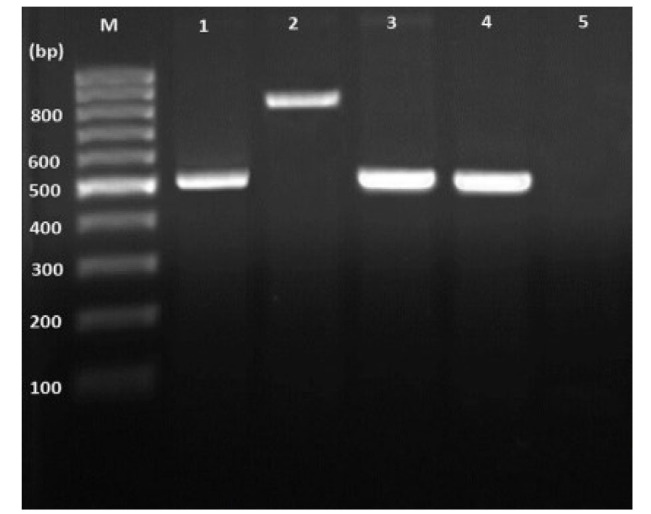
Electrophoretic results of 21-plex PCR assay primers on agarose gel 1%. 1. *Candida albicans*, 2. *Candida dubliniensis*, 3 and 4. *Candida parapsilosis*, M -marker bp50

In brief, PCR amplification was performed in a total reaction volume of 50 μL. Each reaction contained 0.5 μL of forward and reverse primers (25 μM each) [ 12 ], 5 μL of 10X PCR buffer master mix (Yekta Tajhiz Azma, Iran), 1 μL of 50 mM MgCl₂, 0.5 μL of 10 mM dNTPs (each), 0.5 μL of Taq DNA polymerase (5 U/μL), 2 μL of DNA template, and deionized distilled water added to reach the final volume. The PCR reactions were carried out using a thermal cycler (Peqlab, Germany). The PCR thermal cycling conditions were as follows: an initial denaturation at 95 °C for 6 min, followed by 35 amplification cycles consisting of denaturation at 95 °C for 30 sec, annealing at 60 °C for 45 sec, and extension at 72 °C for 60 sec, concluding with a final extension step at 72 °C for 7 min. Integrity and size of the PCR products were subsequently assessed via electrophoresis on a 1% agarose gel, visualized using the Gel Doc XR imaging system (Bio-Rad, USA) in conjunction with a smart ladder (Yekta Tajhiz Azma, Iran) (Figure 1). The DNA from *Candida albicans* ATCC 10231 at a concentration of 10 ng/μL functioned as a positive control, whereas sterile distilled water was employed as the negative control. Thirteen PCR products exhibiting successful amplification were selected for sequencing of the panfungal ITS1-5.8S-ITS2 region. 

### In vitro antifungal susceptibility testing

The AFSTs of fluconazole (FLC) (Sigma-Aldrich, USA), amphotericin B (AmB) (Sigma-Aldrich, USA), and caspofungin (CSP) (Sigma-Aldrich, USA) were performed as the CLSI recommendations (CLSI M27-A3) [ 23 ]. The MIC values were interpreted according to the CLSI M27-A3 and M27-S4 clinical breakpoints documents [ 24 , 25 ]. The CSP, AmB, and FLC serial concentrations were 0.007-8 µg/mL, 0.032-16 µg/mL, and 0.06-64 µg/mL, respectively. For AFST, inocula and cell suspensions were prepared using a spectrophotometric method, measured at 530 nm. The suspensions were subsequently diluted at a ratio of 1:1000 in RPMI 1640 medium (Gibco, UK), buffered to pH 7.0 with 0.165 M 3-N-morpholinopropane sulfonic acid, and adjusted to a final concentration ranging from 1 × 10^3^ to 5 × 10^3^ CFU/mL. Columns of 96-well plates were filled with 100 µL of each serial dilution. Two columns were designed as positive and negative controls. Positive control had no antifungal agent, and negative control had no fungal inoculum. The 96-well microdilution plates were incubated at 35 °C for 24 and 48 h. Following incubation, visual checks were performed to assess antifungal activity, using *C. parapsilosis* (ATCC 22019) and *C. krusei* (ATCC 6258) as quality control strains in all experiments.

### Statistical analysis

Statistical analyses were performed using the SPSS software (version 26.0). To analyze the qualitative variables (frequency and relative frequency), the Kolmogorov-Smirnov test was first applied to measure the normality of the distribution of the investigated variables. If the variables had a normal distribution, the independent sample t-test was applied to compare the means of each quantitative variable. If the normality was not established, the Mann-Whitney U test was used. The Chi-square or Fisher's exact test was applied to compare the quantitative variables (mean and standard deviation). Statistical analysis of the data was considered significant at a level less than 0.05.

## Results

### Characteristics of patients

One hundred clinical samples were evaluated from 100 patients (54% male) aged 20-100 years old. Yeast positivity was established for 70% of the isolates, and 30% had mixed bacterial and yeast positivity. The highest frequency was 43% in the age group of 60-80 years old, followed by 38% in the age group of 80-100 years old. Moreover, the lowest frequency of positivity was n=5 (in the age group of 20-40 years old), followed by n=14 (in the age group of 40-60 years old (Table 1). The main underlying diseases were chronic obstructive pulmonary disease (COPD) (26%), diabetes (24%), cancer (18%), heart and kidney diseases (12%), and stroke (8%). All patients had urinary tract catheters during hospitalization. Besides, 99% of the patients used antibiotics, and 1% used antifungals before hospitalization. COVID-19 was the most common cause of hospitalization (25%), followed by pneumonia (19%), sepsis (18%), primary urinary infection (16%), decreased level of consciousness (13%), drug overdose (6%), and stroke (3%). Moreover, fever (52%), weakness and lethargy (51%), nausea (47%), vomiting (43%), frequent urination (30%), anorexia (18%), abdominal pain (16%), and cough (2%) were the most clinical symptoms of the patients studied. However, 12% of patients had no clinical symptoms. The ICU was the most prevalent inpatient ward (32%), followed by internal (18%), emergency (17%), surgery (14%), neuroscience (12%), infectious diseases (5%), and cardiovascular (2%) wards. It should be mentioned that 40% of patients had a hospitalization length of 7-14 days, 38% more than 14 days, and 22% less than 7 days. Finally, mortality was reported for 44% of patients, and 56% had a relative recovery (Table 1).

**Table 1:** Patient characteristics and key findings of the study.

Characteristics		*C. albicans*	*C. tropicalis*	*C. glabrata*	*C. parapsilosis*	*C. krusei*	*C. dubliniensis*	*C. kefyr*
**Prevalence** (approved by multiplex assay and reference sequencing)		70(70%)	11(11%)	9(9%)	5(5%)	3(3%)	1(1%)	1(1%)
Gender (*p*=0.011)	Men (n=54)	37(68.5%)	10(18.5%)	2(3.7%)	4(7.4%)	0	1(1.9%)	0
	Women (n=46)	33(71.7%)	1(2.2%)	7(15.2%)	1(2.2%)	3(6.5%)	0	1(2.2%)
Age groups (years) (*p*=0.001)	20-40 (n=5)	2(40%)	0	0	0	2(40%)	0	1(20%)
	40-60(n=14)	8(51.7%)	2(14.3%)	3(21.4%)	1(7.1%)	0	0	0
	60-80(n=43)	32(74.4%)	4(9.3%)	3(7%)	2(4.7%)	1(2.3%)	1(2.3%)	0
	80-100(n=38)	28(73.7%)	5(13.2%)	3(7.9%)	2(5.3%)	0	0	0
Underlying diseases (*p*=0.139)	COPD(n=26)	22(84.6%)	2(7.7%)	1(3.8%)	0	1(3.8%)	0	0
	Diabetes(n=24)	18(75%)	0	3(12.5%)	1(4.2%)	1(4.2%)	1(4.2%)	0
	Cancer(n=18)	9(50%)	6(33.3%)	2(11.1%)	1(5.6%)	0	0	0
	Heart diseases(n=12)	6(50%)	3(25%)	1(8.3%)	2(16.7%)	0	0	0
	Kidney diseases(n=12)	9(75%)	0	1(8.3%)	0	1(3.8%)	0	1(8.3%)
	Stroke(n=8)	6(75%)	0	1(12.5%)	1(12.5%)	0	0	0
Treatment status (*p*=0.999)	Antibiotic(n=99)	69(69.7%)	11(11.1%)	9(9.1%)	5(5.1%)	3(3.3%)	1(1%)	1(1%)
	Antifungal(n=1)	1(100%)	0	0	0	0	0	0
Cause of hospitalization (*p*=0.421)	COVID-19(n=25)	17(68%)	6(24%)	1(4%)	1(4%)	0	0	0
	Pneumonia(n=19)	11(57.9%)	3(15.8%)	3(15.8%)	0	1(5.3%)	1(5.3%)	0
	Sepsis(n=18)	12(66.7%)	2(11.1%)	2(11.1%)	2(11.1%)	0	0	0
	PUI(n=16)	13(81.3%)	0	1(6.3%)	0	1(6.3%)	0	1(6.3%)
	DLC(n=13)	10(76.9%)	0	2(15.4%)	1(7.7%)	0	0	0
	Drug overdose(n=6)	5(83.3%)	0	0	0	1(16.7%)	0	0
	Stroke (n=3)	2(66.7%)	0	0	1(33.3%)	0	0	0
Clinical signs and symptoms (*p*=0.032)	Fever(n=52)	34(66.7%)	9(17.6%)	4(7.8%)	2(3.9%)	1(2%)	0	1(2%)
	Weakness and lethargy (n=51)	35(67.3%)	8(15.4%)	6(11.5%)	2(3.8%)	1(1.9%)	0	0
	Nausea(n=47)	31(66%)	7(14.9%)	3(6.3%)	2(4.3%)	2(4.3%)	1(2.1%)	1(2.1%)
	Vomiting(n=43)	30(69.8%)	5(11.6%)	3(7%)	2(4.7%)	2(4.7%)	1(2.3%)	0
	Frequent urination (n=30)	21(70%)	3(10%)	2(5.7%)	1(3.3%)	1(3.3%)	1(3.3%)	1(3.3%)
	Anorexia(n=18)	11(61.1%)	4(22.2%)	1(5.6%)	1(5.6%)	1(5.6%)	0	0
	Abdominal pain(n=16)	9(56.3%)	2(12.5%)	2(12.5%)	0	2(12.5%)	1(6.2%)	0
	No signs and symptoms (n=12)	N/A	N/A	N/A	N/A	N/A	N/A	N/A
	Cough (n=2)	0	0	1(50%)	1(50%)	0	0	0
Hospital wards (*p*=0.188)	ICU (n=32)	28(87.5%)	1(3.1%)	2(6.3%)	1(3.1%)	0	0	0
	Internal (n=18)	11(61.1%)	4(22.2%)	2(11.1%)	1(5.6%)	0	0	0
	Emergency(n=17)	8(47.1%)	3(17.6%)	2(11.8%)	0	3(17.6%)	0	1(5.6%)
	Surgery (n=14)	10(71.4%)	1(7.1%)	2(14.3%)	0	0	1(7.1%)	0
	Neuroscience(n=12)	8(66.7%)	1(8.3%)	1(8.3%)	2(16.7%)	0	0	0
	Infectious diseases(n=5)	3(60%)	1(20%)	0	1(20%)	0	0	0
	Cardiovascular(n=2)	2(100%)	0	0	0	0	0	0
Hospitalization length (*p*=0.026)	Less than 7 days(n=22)	14(63.6%)	0	5(22.7%)	1(4.5%)	1(4.5%)	0	1(4.5%)
	7 to 14 days (n=40)	25(64%)	9(23.1%)	1(2.6%)	2(5.1%)	2(5.1%)	0	0
	More than 14 days (n=38)	31(79.5%)	2(5.1%)	3(7.7%)	2(5.1%)	0	1(2.6%)	0
Outcome (*p*=0.551)	Mortality (n=44)	30(70.5%)	5(11.4%)	5(11.4%)	2(4.5%)	0	1(2.3%)	0
	Recovery (n=56)	39(69.6%)	6(10.7%)	4(7.1%)	3(5.4%)	3(5.4%)	0	1(1.8%)

**Abbreviations:** COPD: chronic obstructive pulmonary disease, PUI: primary urinary infection, DLC: decreased level of consciousness, ICU: intensive care unit.

### 21-plex PCR assay

Based on the molecular identification carried out in this study, candiduria was caused by *C. albicans* species (70%) and NAC species (30%), including *C. tropicalis* (11%), *C. glabrata* (9%), *C. parapsilosis* (5%), *C. krusei* (3%), *C. dubliniensis* (1%), and *C. kefyr* (1%). These results were supported by the reference sequencing method (Table 1; Figure 1). Results of molecular sequencing were aligned via the online BLAST tool and submitted to GenBank (accession numbers PV595928 to PV595940).

### Patient characteristics according to the results of the molecular assay

The chi-square test showed that the patients aged 60-80 years old were the most frequent carriers of *C. albicans* isolates (*p*=0.001) (Table 1). Prevalence of *C. albicans* was 71.7% among women (*p*=0.011). It was also revealed that patients with COPD and diabetes had the most positivity for *C. albicans*, at 84.6% and 74.1%, respectively. Nonetheless, the observed difference did not reach statistical significance (*p*=0.139). Antibiotic recipients had the highest prevalence rate for *C. albicans* (69.7%) (*p*=0.99). Among patient groups, COVID-19 patients had the highest prevalence of *C. albicans* (24.28%), followed by patients with urinary infections (18.57%). Nonetheless, this comparison was not statistically significant (*p*=0.421). In addition, 35 isolates of *C. albicans* (50%) were obtained from patients with weakness and lethargy (*p*=0.032). Patients from ICUs had the highest prevalence rate for *C. albicans* isolates (40%) (*p*=0.188). Frequency of candidiasis caused by *C. albicans* species was over 79.5% in patients hospitalized for more than 14 days, compared to 36.4% for candidiasis caused by NAC species in patients hospitalized for less than 7 days (*p*=0.026). Finally, the frequency of candidiasis caused by *C. albicans* species in deceased patients was 70.5% (*p*=0.551) (Table 1).

### Antifungal susceptibility testing

The MIC breakpoints/ranges of FLC, CSP, and AmB were defined and interpreted according to the CLSI-M27-A3 recommendation and presented in Table 2. The highest MIC range (64-0.125 μg/mL) belonged to FLC, and the lowest MIC range (8-0.016 μg/mL) belonged to CSP. *Candida tropicalis*, *C. glabrata*, *C. krusei*, and *C. kefyr* had higher MIC ranges of azole drugs (especially FLC), compared to other species. Moreover, MIC ranges of *C. parapsilosis*, *C. glabrata*, and *C. tropicalis* species were higher than those of other species and lower than that of *C. albicans* to CSP.

**Table 2:** Results of antifungal susceptibility tests.

Candida species	MIC (μg/mL)	MIC range (μg/mL)	Susceptibility status		
			Susceptible	(Dose-dependent Or Intermediate)	Resistant
***C. albicans***	FLC	0.125–64	≤2(n=67; 95.71%)	4(n=1; 1.43%)	≥8(n=2; 2.86%)
	AmB	0.062–16	0.25(n=35; 50%)	-(n=35; 50%)	≥2(n: 0)
	CSP	0.032–8	≤0.25(n=65; 2.86%)	0.5(n=3; 4.29%)	≥1(n=2; 2.86%)
***C. tropicalis***	FLC	4–64	≤4(n=6; 54.55%)	8(n=3; 27.27%)	≥16(n=2; 18.18%)
	AmB	0.5–1	≤1 (n=10; 90.91%)	-(n=0)	>2(n=1; 9.09%)
	CSP	0.032–2	≤0.25(n=8; 72.73%)	0.5(n=2; 18.18%)	≥1(n=1; 9.09%)
***C. glabrata***	FLC	0.25–2	≤16(n=4; 44.44%)	32(n=3; 33.33%)	≥64(n=2; 22.22%)
	AmB	0.25–1	≤1(n=8; 88.89%)	-(n=1; 11.11%)	>2(n: 0)
	CSP	0.125–2	≤0.012(n: 0)	0.25(n=3; 33.33%)	≥0.5(n=6;66.67%)
***C. parapsilosis***	FLC	0.125–64	≤2(n=5; 100%)	4(n=0)	≥8(n: 0)
	AmB	0.06–1	≤1(n=4; 80%)	-(n=0)	≥1(n=1; 20%)
	CSP	1–8	≤2(n=2; 40%)	4(n=2; 40%)	≥8(n=1; 20%)
***C. krusei***	FLC	0.5–8	≤8(n=1; 33.33%)	16–32(n=2; 66.67%)	≥64(n: 0)
	AmB	0.25–4	≤1(n=1; 33.33%)	-(n=0)	>2(n: 2; 66.67%)
	CSP	0.25–2	≤0.012(n=3; 100%)	0.25(n=0)	≥0.5(n=0)
***C. dubliniensis***	FLC	0.125–8	0.125(n: 0)	0.5(n=0)	≤1(n: 1; 100%)
	AmB	0.25–4	≤0.031(n: 0)	0.25(n=1; 100%)	≥2(n=0)
	CSP	0.25–2	≤0.016(n=1; 100%)	0.125(n: 0)	≥1(n=0)
***C. kefyr***	FLC	0.5–8	≤8(n=0)	16–32(n=1; 100%)	≥64(n=0)
	AmB	0.25–4	≤1(n=1; 100%)	-(n=0)	>2(n=0)
	CSP	0.25–2	≤2(n=1; 100%)	-(n=0)	>2(n=0)

**Abbreviations:** MIC: Minimum inhibitory concentration, FLC: Fluconazole, AmB: Amphotericin B, CSP: Caspofungin.

Results of AFST are presented by frequency of 100 isolates (Table 2). Among 70 *C. albicans* isolates, 67 (95.71%) and 65 (92.86%) were susceptible to FLC and CSP, respectively. In contrast, 24 (27.27%) isolates were susceptible to AmB in NAC species. Moreover, two *C. albicans* isolates (2.86%) were resistant to FLC and CSP, in contrast to NAC species, 5 of which (29.41%) were resistant to FLC and 8 (47.05%) of which were resistant to CSP.

One of three (33.33%) *C. krusei* isolates, 1 (100.0%) *C. kefyr* isolate, 10 of 11 (90.91%) *C. tropicalis* isolates, 8 of 9 (88.89%) *C. glabrata* isolates, and 4 of 5 (80.0%) *C. parapsilosis* isolates were susceptible to AmB. In addition, all *C. krusei* isolates (100%) and 8 of 11 (72.83%) *C. tropicalis* isolates were susceptible to CSP. One (100.0%) *C. dubliniensis* isolate was resistant to FLC, and 2 (66.67%) *C. krusei* isolates were resistant to AmB. Meanwhile, 1 (20%) *C. parapsilosis* was resistant to each AmB and CSP. Moreover, *C. glabrata* showed resistance to CSP and FLC with 6 (66.67%) and 2 (22.22%) isolates, respectively. Two (18.18%) *C. tropicalis* isolates were also resistant to FLC, AmB, and CSP. The single isolate of *C. kefyr* was resistant to CSP and AmB (Table 2).

## Discussion

Prevalence of iatrogenic candidiasis has been elevated during the last three decades [ 2 , 26 ]. Candiduria is one of the most common clinical manifestations of urinary tract fungal infections. Although it is frequently asymptomatic, it may also indicate underlying conditions, such as cystitis, pyelonephritis, prostatitis, epididymitis, or even disseminated candidiasis [ 2 , 26 ]. Emergence of antifungal-resistant species complicates their management. Despite the high rate of complications, the mortality rate is low. *Candida albicans* is recognized as the primary cause of candiduria; however, NAC species are increasing as potential etiologic agents [ 6 ].

In the present study, using a 21-plex-PCR system, it was found that among 100 patients with candiduria, *C. albicans* was the predominant cause (70%), followed by *C. tropicalis* (11%), *C. glabrata* (9%), *C. parapsilosis* (5%), and *C. krusei* (3%). Kord et al. [ 13 ] established a 21-plex PCR system for identifying various yeast species and achieving acceptable results. Based on a review performed by Gharaghani et al. [ 27 ] in Iran, *C. albicans* was the predominant cause of candiduria; however, the prevalence of NAC species, especially *C. parapsilosis*, *C. krusei*, *C. glabrata*, and *C. tropicalis*, was increased. Moreover, they reported that men were more affected than women. Moazeni et al. [ 28 ] in their study concluded that *C. albicans* (59.4%) was the leading cause of candiduria, followed by *C. glabrata* (21.6%), *C. tropicalis* (13.5%), *C. krusei* (4.0%), and *C. parapsilosis* (1.3%). Their findings were consistent with those of most studies conducted in Iran, as well as the present study. However, in some studies, NAC species, especially *C. glabrata*, were the dominant cause of candiduria species [ 29 ]. For instance, Lima et al. in their study [ 30 ]concluded that the prevalence of NAC species was 64.4%, and *Candida tropicalis* was the primary causing agent (39.6%), followed by *C. albicans* (31.1%). This indicated a sharp switch from *C. albicans* to NAC species, possibly due to the broad-spectrum use of novel antifungals in the clinic. However, it was shown that *C. albicans* was the primary cause of candidiasis among patients with malignancy [ 31 ].

In the present study, it was found that the prevalence of candiduria was higher in men (54%). Moreover, it was revealed that the frequency of candiduria had a direct relationship with the age of patients. Accordingly, patients aged 60-80 had the highest frequency (43%). In addition, the ICU ward had the highest prevalence of candiduria among the other wards. It was also concluded that the older patients, due to extended hospitalization, increased use of urinary catheters (especially Foley catheters), and undergoing invasive procedures, are more exposed to candiduria with *C. albicans*, *C. tropicalis*, and *C. glabrata*. Lima et al. [ 30 ] in their research reported that the prevalence of candiduria was higher in women (54.7%), and 65.1% of their patients were older than 60 years. Besides, 33.1% of their patients were from the ICU and emergency wards. Similarly, in the present study, the frequency of *C. albicans* was higher in women (71.7%) and patients 60-80 years old (74.4%)

The COPD (26%) and diabetes (24%) were the main underlying diseases of the patients in this study. Prevalence of *C. albicans* was higher than that of the NAC species and was observed in 84.6% and 75.1% of patients with COPD and diabetes, respectively. However, this was insignificant (*p*=0.139). Mert and Odabasi [ 32 ] reported in a review that candiduria is commonly observed in hospitalized patients, and most patients are asymptomatic.

In the present research, it was found that patients with hospitalization lengths of 7-14 days (40%) and above 14 days (38%) had the highest rate of candiduria. *Candida albicans* was significantly higher than NAC species in both groups (*p*=0.026), which was consistent with the findings of a study performed by Hamory et al. [ 33 ].

In this study, the mortality rate was 44%, and the frequency of *C. albicans* was 70.5% among them; however, it was insignificant (*p*=0.551). Several studies have reported that the mortality of patients with candiduria was more dependent on risk factors and predisposing factors.

COVID-19 was the most prevalent cause of hospitalization in this study (25%), and *C. albicans* was the leading cause of candiduria among them (*p*=0.421). Most cases of candiduria by NAC species were caused by *C. parapsilosis* (33.3%) in hospitalized patients due to stroke, and also *C. tropicalis* (24%) in COVID-19 patients. Hamory et al. [ 33 ] reported that the leading cause of hospitalization in their study was immunosuppression. However, the impact of COVID-19 and secondary fungal infections in our geographical zone was previously highlighted [ 34 ].

In the current study, 99% of patients diagnosed with candiduria had a history of steroid use and broad-spectrum antibiotic administration. Similarly, a study by Guler et al. [ 35 ] reported that prior antibiotic use increased the risk of developing candidiasis by 6-fold. Additionally, 7.8% of candidiasis cases in their cohort involved patients with cancer, with malignancy associated with a 0.2-fold increase in candidiasis risk. Previous research has demonstrated that prolonged antibiotic exposure compromises host immune defenses by diminishing phagocytic function and reducing antibody production, thereby lowering resistance to *Candida* infections [ 36 , 37 ].

Fever, alongside weakness and lethargy, was the most prominent clinical manifestation among the patients of the present study (52% and 51%, respectively). Accordingly, 34% and 35% of *C. albicans* species were isolated from patients with fever and weakness-lethargy. Nevertheless, *C. tropicalis* was the main NAC species among patients with fever (9%) and weakness-lethargy (8%). Bukhary et al. [ 38 ] in their study showed that clinical symptoms in candiduria appear with signs and symptoms of bladder irritation, including heartburn, hematuria, nausea, anorexia, and abdominal pain. *Candida* pyelonephritis can be associated with candidemia, sepsis, and septic shock. It is rarely seen in hospitalized patients with diabetes and renal failure with papillary necrosis and obstructive uropathy, which cannot be distinguished from bacterial pyelonephritis and urosepsis. Candiduria and fever may be the only clues and first signs of systemic invasive candidiasis in high-risk patients, including those with neutropenia and kidney, liver, or bone marrow transplant recipients, as well as people who have undergone invasive urological procedures. They finally concluded that blood culture and radiological investigations are necessary to determine the anatomical source of candidiasis for candiduria patients with fever or sepsis.

Emergence of antifungal resistance is one of the main problems in managing IFIs. In this study, of 70 *C. albicans* species, 67 (95.71%) and 65 (92.86%) isolates were susceptible to FLC and CSP, respectively. In addition, 35 (50%) isolates were dose-dependent susceptible to AmB. Moreover, out of 30 NAC species, 24 (27.27%) and 16 (29.10%) isolates were susceptible to AmB and FLC, and 15 (43.63%) isolates were susceptible to CSP. Two of them (6.66%) were reported to be dose-dependent to AmB. Two (2.86%) of the *C. albicans* species were resistant to FLC and CSP, while 5 (16.6%) NAC species were resistant to FLC, and 8 (26.6%) were resistant to CSP. This shows that the emergence of resistance among NAC species was higher than the *C. albicans* species. Lima et al. [ 30 ] reported the emergence of resistance to several antifungals, especially FLC, by *C. albicans* isolates. However, all *C. albicans* isolates were susceptible to AmB and CSP. In addition, all NAC species were susceptible to the tested antifungals (FLC, AmB, and CSP), except for *C. krusei*, which is intrinsically resistant to FLC.

Several studies carried out in Brazil have reported the emergence of resistant isolates to FLC [ 39 , 40 ], although Tavanti et al. [ 41 ] reported no such resistance. In Iran, Esmailzadeh et al. [ 42 ] reported the high prevalence of candiduria caused by NAC species among diabetic patients. Moreover, they found that the level of azole-resistant *C. albicans* isolates is generally low. However, the prevalence of triazole-resistant NAC species, including *C. krusei* and *C. glabrata*, especially to FLC, was high. This indicates that due to the elevated resistance rate of NAC species to antifungals, accurate identification of *Candida* species, especially in symptomatic candiduria, appears necessary.

One of the limitations of the current study was the lack of positive cases of proven candiduria during the study period. However, this may be due to insufficient knowledge of clinical diagnosis. It was attempted to solve this issue by extending the study period and incorporating additional clinical wards. Another limitation was the challenge of distinguishing proven candiduria from *Candida* colonization. In this regard, the clinical signs and symptoms of candiduria were considered as discussed in the methods section. 

## Conclusion

Findings of this study confirm that *Candida albicans* remains the predominant etiological agent of candiduria; nonetheless, NAC species exhibited a higher resistance rate to FLC, compared to *C. albicans*. It was shown that our multiplex system was successful in determining the candiduria agents. Since the emergence of antifungal-resistant species is an important challenge of IFI management, future molecular investigations employing innovative and diverse methodologies, along with larger and more representative patient populations, are recommended to enhance and expand the current understanding in this field. 
